# Strain Localization of Elastic-Damaging Frictional-Cohesive Materials: Analytical Results and Numerical Verification

**DOI:** 10.3390/ma10040434

**Published:** 2017-04-20

**Authors:** Jian-Ying Wu, Miguel Cervera

**Affiliations:** 1 State Key Laboratory of Subtropical Building Science, South China University of Technology, Guangzhou 510641, China; 2 CIMNE, Technical University of Catalonia, Edificio C1, Campus Norte, Jordi Girona 1-3, 08034 Barcelona, Spain; miguel.cervera@upc.edu

**Keywords:** localized failure, strain localization, damage, frictional-cohesive materials, constitutive behavior

## Abstract

Damage-induced strain softening is of vital importance for the modeling of localized failure in frictional-cohesive materials. This paper addresses strain localization of damaging solids and the resulting consistent frictional-cohesive crack models. As a supplement to the framework recently established for stress-based continuum material models in rate form (Wu and Cervera 2015, 2016), several classical strain-based damage models, expressed usually in total and secant format, are considered. Upon strain localization of such damaging solids, Maxwell’s kinematics of a strong (or regularized) discontinuity has to be reproduced by the inelastic damage strains, which are defined by a bounded characteristic tensor and an unbounded scalar related to the damage variable. This kinematic constraint yields a set of nonlinear equations from which the discontinuity orientation and damage-type localized cohesive relations can be derived. It is found that for the “Simó and Ju 1987” isotropic damage model, the localization angles and the resulting cohesive model heavily depend on lateral deformations usually ignored in classical crack models for quasi-brittle solids. To remedy this inconsistency, a modified damage model is proposed. Its strain localization analysis naturally results in a consistent frictional-cohesive crack model of damage type, which can be regularized as a classical smeared crack model. The analytical results are numerically verified by the recently-proposed mixed stabilized finite element method, regarding a singly-perforated plate under uniaxial tension. Remarkably, for all of the damage models discussed in this work, the numerically-obtained localization angles agree almost exactly with the closed-form results. This agreement, on the one hand, consolidates the strain localization analysis based on Maxwell’s kinematics and, on the other hand, illustrates versatility of the mixed stabilized finite element method.

## 1. Introduction

It is well known that under certain circumstances, frictional-cohesive materials with a softening regime exhibit strain localization prior to the occurrence of macroscopic failure. For instance, in the so-called frictional $J_{2}$ (von Mises) materials, the shear (or slip) strains usually concentrate, leading to the formation of shear bands with a small, but finite width or slip lines with a vanishing width. Similarly, cohesive crack bands or surfaces are often observed in frictional-cohesive geomaterials like concrete and rocks. Once such strain localization occurs, inside and outside these domains with highly localized deformations, the strain fields are either discontinuous due to the continuous, but non-smooth displacements, or even singular (unbounded), caused by the discontinuous displacements. These strain (or weak) and displacement (or strong) discontinuities are usually regarded as prognostics of localized failure in frictional-cohesive materials and resulting catastrophic collapse of engineering structures. Therefore, it is of vital importance to predict the occurrence of strain localization and quantify its adverse influence on the overall response.

In the theoretical context, strain localization and the induced localized failure in frictional-cohesive materials can be characterized by either generalized continuum models or nonlinear crack/fracture models. In the former approach, the effects of strain/displacement discontinuities are smoothed or smeared. Accordingly, the overall nonlinear behavior of the weakened material can be described by a tensorial constitutive law in terms of stress vs. strain. Among many alternatives, plasticity [1] and damage mechanics [2] or their combination [3,4,5] are frequently employed to develop appropriate inelastic constitutive laws, usually based on the irreversible thermodynamics with internal variables. Comparatively, in nonlinear crack/fracture models, displacement jumps are explicitly accounted for by embedding the discontinuities into a solid matrix (bulk) along preferred orientations. It is in general assumed that the discontinuity in which energy dissipation localizes is characterized by a vectorial traction-based frictional-cohesive zone model while the bulk remains elastic, between which the traction continuity condition is imposed. Similarly, depending on the recoverable/irreversible properties of the discontinuities, frictional-cohesive zone models of either the plastic [6], damage [7,8,9] or combined plastic-damage [10,11] type can be established. Note that in both approaches, the introduction of fracture energy per discontinuity surface, usually regarded as a material property, is indispensable to guarantee the objectivity of energy dissipation during the whole failure process [12,13].

Though both the continuum and crack/fracture models are able to quantify the overall effects of localized failure in frictional-cohesive materials, their theoretical fundamentals and physical motivations are rather distinct from each other. It is of great significance to investigate and clarify the interrelations between them. On the one hand, the constitutive relations of a frictional-cohesive zone model, in terms of tractions versus displacement jumps (or the regularized inelastic deformation vector), are generally established in a heuristic or ad hoc manner. This heavily restrains developing a physically-sound and theoretically-rational cohesive zone model for mixed-mode failure. On the other hand, the smeared crack model can be regarded as a particular generalized continuum model in between both families. More specifically, it can be recovered by combining of the elastic bulk and the nonlinear cohesive discontinuity upon the assumption of a continuous stress field overall the whole solid; see [14,15]. Therefore, it would be rather enlightening if the above procedure could be performed inversely, i.e., developing a frictional-cohesive zone model in the context of discrete crack methods [16,17] from its continuum counterpart with softening material laws.

Employing the strong discontinuity approach [18,19], Oliver et al. [20,21,22,23,24] derived frictional-cohesive zone models by projecting inelastic material laws onto the discontinuity orientation. Similar work has also been done in [25,26]. However, only very simple continuum models, e.g., the classical isotropic damage model [22,23,24], the Rankine and plane strain $J_{2}$ (von Mises) plasticity models [20,21], can be considered, whereas more general material constitutive laws cannot be sufficiently accounted for [21]: “obtaining such explicit forms of the discrete constitutive equations is not so straight-forward for other families of elastoplastic models”. Furthermore, as the discontinuity orientation is determined from the discontinuous bifurcation condition [27,28,29,30,31,32] together with the null softening modulus, strong (regularized) discontinuities cannot form in general cases. Consequently, some kinematic mismatches are observed [24,33], resulting in spurious stress locking [34,35].

To overcome the issue of mispredicted discontinuity orientation, Cervera et al. [34] suggested directly using Maxwell’s kinematic conditions of strong (or regularized) discontinuities to determine the discontinuity orientation, so that the stress locking-free property can be guaranteed for a fully-softened discontinuity. The closed-form results of the localization angles for the $J_{2}$ plasticity model were validated by numerical simulations in the cases of plane stress and plane strain. More recently, the authors [15,36] successfully extended the above strain localization analysis to a unified stress-based plastic-damage model with general (e.g., Rankine, von Mises, Mohr–Coulomb, Drucker–Prager and more complex elliptic, parabolic, hyperbolic, etc.) failure criteria. Both general 3D and 2D (plane stress and plane strain) cases were considered. Not only the discontinuity orientation, but also the corresponding cohesive zone model, i.e., constitutive relations, evolution equations, traction-based failure criterion, softening functions, etc., are determined consistently from the given stress-based counterpart. Furthermore, the bi-directional connections and in particular the equivalence conditions between two complementary methodologies for the modeling of localized failure in quasi-brittle solids, i.e., traction-based discontinuities localized in an elastic solid and strain localization of a stress-based inelastic softening solid, have also been fully established. Numerical results obtained from the stress accurate stabilized mixed elements [34,37] coincide with the theoretical predictions, validating the developed framework.

On the one hand, so far, Maxwell’s kinematics-based strain localization has been employed mainly to analyze stress-based elastoplastic damage models with the inelastic strain expressed in rate form. Consequently, in general load scenarios, the normal strains not acting on the discontinuity surface, caused by Poisson’s lateral effects, can vanish in such a way that Maxwell’s kinematics of strong (or regularized) discontinuities is accommodated upon strain localization. On the other hand, the continuum damage model, usually expressed in the strain-based total form, has also been widely accepted as an alternative to deal with complex material behavior. The application includes, but is not limited to, creep, fatigue and other nonlinear behavior of frictional-cohesive materials; see [38] for a review. The reason for its popularity is as much the intrinsic simplicity and versatility of the theory, as well as its consistency based on the irreversible thermodynamics with the internal variable. However, it is not so straightforward to extend the aforesaid Maxwell’s kinematics-based strain localization to strain-based continuum models, since the resulting inelastic (damage) strain depends heavily on the lateral deformations induced by Poisson’s ratio. This fact is inconsistent with the frictional-cohesive zone models, which generally neglect the strain and stress triaxiality. As will be shown, owing to this conceptual contradiction, strain localization of a general strain-based isotropic damage model cannot always be guaranteed as of stress-based ones.

This paper addresses the above challenging issue. Its objectives are four-fold: (i) to present strain localization analysis of strain-based isotropic damage models in a unified manner; (ii) to analyze strain localization of two classical damage models and, in particular, to derive closed-form results of the discontinuity orientation and corresponding localized frictional-cohesive model; (iii) to advocate a new modified damage model for frictional-cohesive materials, with its localized counterpart consistent with the assumption of classical discrete and smeared crack models; and (iv) to numerically validate the analytical results by the mixed stabilized finite element method [39,40].

The structure of this paper is outlined as follows. After this Introduction, Maxwell’s kinematics-based strain localization analyses of strain-based isotropic damage models are presented in Section 2 in a unified approach. In Section 3, the classical $J_{2}$ and Simó and Ju [41] damage models, as well as a new modified Simó and Ju [41] model are analytically investigated. In particular, the closed-form results of the localization angles and the corresponding localized frictional-cohesive models are derived consistently. Section 4 addresses the numerical verification of the proposed strain localization analysis, regarding numerical simulations of a singly-perforated plate under uniaxial tension. The effects of Poisson’s ration on the localization angles are investigated. The most relevant conclusions are drawn in Section 5. Finally, two appendices are attached to close this paper.

Notation: Compact tensor notation is used in this paper. As a general rule, scalars are denoted by italic light-face Greek or Latin letters (e.g., *a* or λ); vectors and second-order tensors are signified by italic boldface minuscule and majuscule letters like a and A, respectively. Fourth-order tensors are identified by blackboard-bold majuscule characters (e.g., A). Symbols I and I represent the second-order and symmetric fourth-order identity tensors, respectively. Superscripts ‘${}^{T}$’ and ‘${}^{\mathrm{sym}}$’ indicate the transposition and symmetrization operations, respectively. The inner products with single and double contractions are denoted by ‘·’ and ‘:’, respectively. The dyadic product ‘⊗’ and the symmetrized Kronecker product $\;\underline{\bar{⊗}}\;$ are defined as:

$${\left(A⊗B\right)}_{ijkl}=A_{ij}B_{kl},\;{\left(A\;\underline{\bar{⊗}}\;B\right)}_{ijkl}=\frac{1}{2}\left(A_{ik}B_{jl}+A_{il}B_{jk}\right)$$

## 2. Strain Localization in Damaging Solids

### 2.1. Continuum Damage Models

For a damaging solid, the constitutive relation between the second-order stress σ and (infinitesimal) strain ϵ is expressed as:

(1) $$\begin{array}{c} \sigma =E:\epsilon ,\;\epsilon =C:\sigma \end{array}$$

where E and $C:=E^{-1}$ represent the fourth-order (variable) secant stiffness and compliance tensors, respectively.

**Figure 1:**
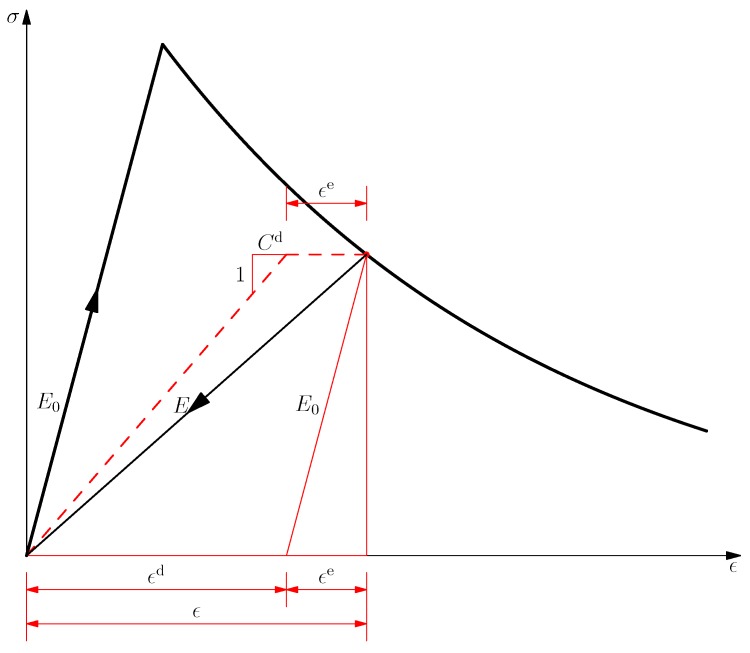
Elastic-damage model in the 1D case.

As depicted in Figure, the strain tensor ϵ can be kinematically decomposed into the following additive form:

(2) $$\begin{array}{c} \epsilon =\epsilon^{e}+\epsilon^{d} \end{array}$$

where the elastic strain $\epsilon^{e}$ and the damage one $\epsilon^{d}$, both being recoverable upon unloading, are given by:

(3) $$\begin{array}{c} \epsilon^{e}=C_{0}:\sigma ,\;\epsilon^{d}=C^{d}:\sigma \end{array}$$

for the damage compliance tensor $C^{d}:=C-C_{0}$ defined as the difference of the total secant one C with respect to the undamaged elasticity one $C_{0}$. The above kinematic decomposition is depicted in Figure 1 for the 1D case.

In the above constitutive relations, the damage compliance $C^{d}$ (or, equivalently, the secant compliance C or stiffness E) is an internal variable. Based on different approximations, either isotropic, orthotropic or fully-anisotropic evolution laws can be postulated for the fourth-order damage compliance $C^{d}$; see [3,11,36,42,43,44] for more details. Among them, due to the conceptual simplicity, the strain-based 1−d damage model, with d∈[0,1] representing the single damage scalar index, has been widely adopted in the literature. For such a model, as will be shown later, the damage compliance can be expressed in the following total format:

(4) $$\begin{array}{c} C^{d}=\frac{d}{1-d}{\bar{C}}^{d}=\omega {\bar{C}}^{d}\;⟹\;\epsilon^{d}={\bar{C}}^{d}:\sigma =\omega \Lambda \end{array}$$

where ω:=d/(1−d) is an alternative damage variable in the range [0,∞]; the fourth-order tensor ${\bar{C}}^{d}$ and second-order one $\Lambda :={\bar{C}}^{d}:\sigma$, both being model-dependent and bounded, characterize the damage compliance $C^{d}$ and strain $\epsilon^{d}$, respectively.

**Figure 2:**
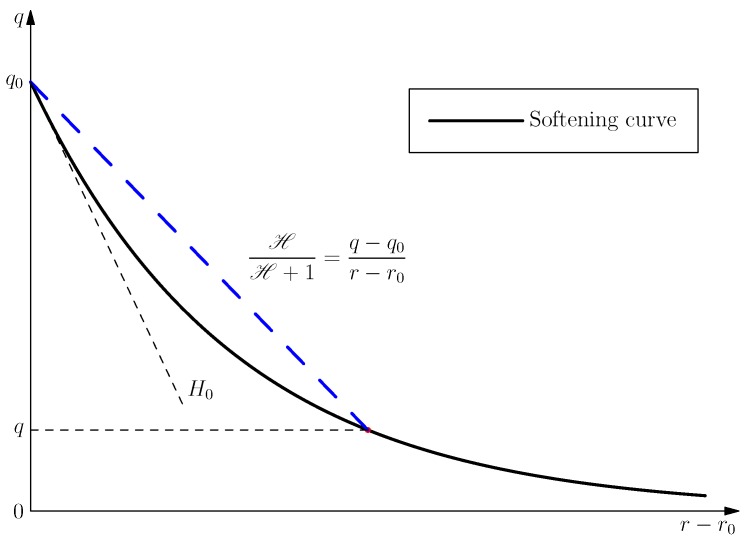
Definition of the continuum softening parameters *H* and H.

With the above definitions, only the evolution law for the damage scalar *d*, rather than for the complicate tensor $C^{d}$, needs to be postulated. Without loss of generality, this can be expressed as:

(5) $$\begin{array}{c} d\left(r\right)=1-\frac{q}{r},\;q=\hat{q}\left(r\right)=\left(1-d\right)r \end{array}$$

where the stress-like internal variable $\hat{q}\left(r\right)$ is a monotonically-decreasing function of the history variable (threshold) *r*, with identical initial value $q_{0}=r_{0}$. For instance, the linear function:

(6a) $$\begin{array}{c} \hat{q}\left(r\right)=\left\{\begin{array}{cc} \left[1+H\cdot \left(\frac{r-r_{0}}{r_{0}}\right)\right]q_{0} & \;r_{0}\leq r\leq r_{u}\\ 0 & \;r\geq r_{u} \end{array}\right. \end{array}$$

or the exponential one:

(6b) $$\begin{array}{c} \hat{q}\left(r\right)=q_{0}\exp\left[H\cdot \left(\frac{r-r_{0}}{r_{0}}\right)\right] \end{array}$$

is frequently adopted, where the parameter H<0 controls the softening function $\hat{q}\left(r\right)$; see Figure 2.

Let us introduce the secant slope $H_{s}$ of the *q* versus $r-r_{0}$ softening curve, so that:

(7) $$\begin{array}{c} H_{s}:=\frac{q-q_{0}}{r-r_{0}}=\frac{H}{H+1}<0\;⟺\;r-q=\frac{1}{H}\left(q-q_{0}\right) \end{array}$$

Note that the secant value $H_{s}<0$ is not coincident with the softening parameter *H* introduced in Equation (6); only for the linear softening case, the relation $H=H_{s}$ holds; see Figure 2 for the illustration.

Accordingly, the alternative damage variable ω can be expressed as:

(8) $$\begin{array}{c} \omega :=\frac{d}{1-d}=\frac{r-q}{q}=\frac{1}{H}\left(1-\frac{q_{0}}{q}\right) \end{array}$$

which results in the following damage strain tensor $\epsilon^{d}$:

(9) $$\begin{array}{c} \epsilon^{d}=C^{d}:\sigma =\omega \Lambda =\frac{1}{H}\left(1-\frac{q_{0}}{q}\right)\Lambda \end{array}$$

Note that the entity $\left(1-q_{0}/q\right)$, related to the stress-like internal variable *q*, and the characteristic tensor Λ, dependent on the stress σ, are both bounded.

### 2.2. Strain Localization and Localized Damage Models

For strain localization to occur in a softening solid and to develop eventually into a fully-softened discontinuity at the final stage of the deformation process, material points inside the strain localization band undergo inelastic loading, while those outside it unload elastically [20,21,34]. As recently clarified by the authors [45], this standpoint is equivalent to strong discontinuities of the displacement field localized in an elastic solid. Based on this equivalence, novel strain localization analysis [15,36] based on the satisfaction of Maxwell’s kinematics [32] and of stress continuity has been proposed. Particularly, for a stress-based material model with a softening regime, not only the discontinuity (band) orientation, but also the corresponding traction-based cohesive model can be consistently derived. In this section, the aforesaid method is extended to the strain-based damage models addressed in Section 2.1.

**Figure 3:**
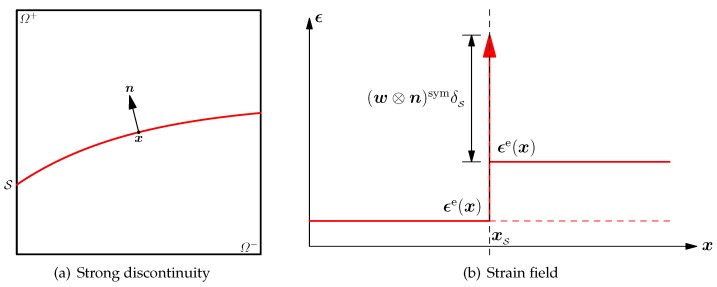
Strong discontinuity and the resulting singular strain field in an elastic solid.

Let us first consider an elastic solid Ω containing a strong discontinuity S as depicted in Figure 3a. The orientation of the discontinuity S is characterized by the normal vector n, as well as two tangential vectors m and p, constituting a local coordinate system (n,m,p). Upon strain localization, the standard kinematics of a continuum medium are replaced by Maxwell’s kinematics [32], which allows for jumps in the derivatives of the displacement field with respect to the direction normal to the discontinuity S, but not in the derivatives with respect to the direction tangential to it. According to the above Maxwell kinematics, the jump in the strain field between the inside and the outside of the localization band may be expressed exclusively in terms of the unit normal vector and a deformation vector. Therefore, the singular strain field ϵ caused by the displacement jump 〚u〛=w across the discontinuity S is expressed as:

(10) $$\begin{array}{c} \epsilon =\epsilon^{e}+{\left(w⊗n\right)}^{\mathrm{sym}}\;\delta_{{}_{S}} \end{array}$$

where $\epsilon^{e}$ represents the bulk strain field, being elastic but not necessarily continuous across the discontinuity S; the Dirac-delta $\delta_{{}_{S}}$ is introduced to characterize the singular strain field at the interface S; see Figure 3b.

Recalling the aforementioned equivalence, for strain localization in damaging solids to occur, it follows from Maxwell’s kinematic (10) that:

(11) $$\begin{array}{c} \epsilon^{d}=C^{d}:\sigma =\omega \Lambda ={\left(w⊗n\right)}^{\mathrm{sym}}\;\delta_{{}_{S}} \end{array}$$

As the stress σ and the resulting characteristic tensor Λ are both bounded, upon strain localization, the damage compliance $C^{d}$ and the variable ω in Equation (9) have to be singular, i.e.,

(12) $$\begin{array}{c} C^{d}={\bar{C}}^{d}\;\delta_{{}_{S}},\;\omega =\bar{\omega }\;\delta_{{}_{S}} \end{array}$$

with ${\bar{C}}^{d}$ and $\bar{\omega }$ both being bounded.

Calling for the relation (8), the singularity of ω implies the existence of a bounded softening parameter $\bar{H}<0$, such that:

(13) $$\begin{array}{c} \frac{1}{H}=\frac{1}{\bar{H}}\;\delta_{{}_{S}},\;\bar{\omega }=\frac{1}{\bar{H}}\left(1-\frac{q_{0}}{q}\right) \end{array}$$

It then follows that:

(14) $$\begin{array}{c} \omega \Lambda =\bar{\omega }\Lambda \;\delta_{{}_{S}}={\left(w⊗n\right)}^{\mathrm{sym}}\;\delta_{{}_{S}}\;⟹\;\bar{\omega }\Lambda ={\left(w⊗n\right)}^{\mathrm{sym}} \end{array}$$

Therefore, the displacement jump w can be solved in terms of the characteristic tensor Λ as:

(15) $$\begin{array}{c} w=w_{n}n+w_{m}m+w_{p}p=\bar{\omega }\left(2n\cdot \Lambda -n\Lambda_{nn}\right) \end{array}$$

where the local components $\left(w_{n},w_{m},w_{p}\right)$ of the displacement jump vector w in the orthogonal system (n,m,p) of the discontinuity S are given by:

(16a) $$\begin{array}{cc} & w_{n}:=w\cdot n=\bar{\omega }\Lambda_{nn},\;w_{m}:=w\cdot m=2\bar{\omega }\Lambda_{nm} \end{array}$$

(16b) $$\begin{array}{c} w_{p}:=w\cdot p=2\bar{\omega }\Lambda_{np} \end{array}$$

Substitution of the above results into the relation (14) yields:

(17a) $$\begin{array}{cc} & \Lambda_{mm}\left(\theta^{\mathrm{cr}}\right)=0 \end{array}$$

(17b) $$\begin{array}{c} \Lambda_{pp}\left(\theta^{\mathrm{cr}}\right)=0,\;\Lambda_{mp}\left(\theta^{\mathrm{cr}}\right)=0 \end{array}$$

for the discontinuity angles $\theta^{\mathrm{cr}}$ upon which the kinematic conditions (14) are satisfied. Calling for the relations of the local components $\left(\Lambda_{mm},\Lambda_{pp},\Lambda_{mp}\right)$ between the principal values $\Lambda_{i}\;\left(i=1,2,3\right)$ and a set of characteristic angles θ, the kinematic constraints (17) yield a system of nonlinear equations, such that the discontinuity angles $\theta^{\mathrm{cr}}$ can be determined.

**Figure 4:**
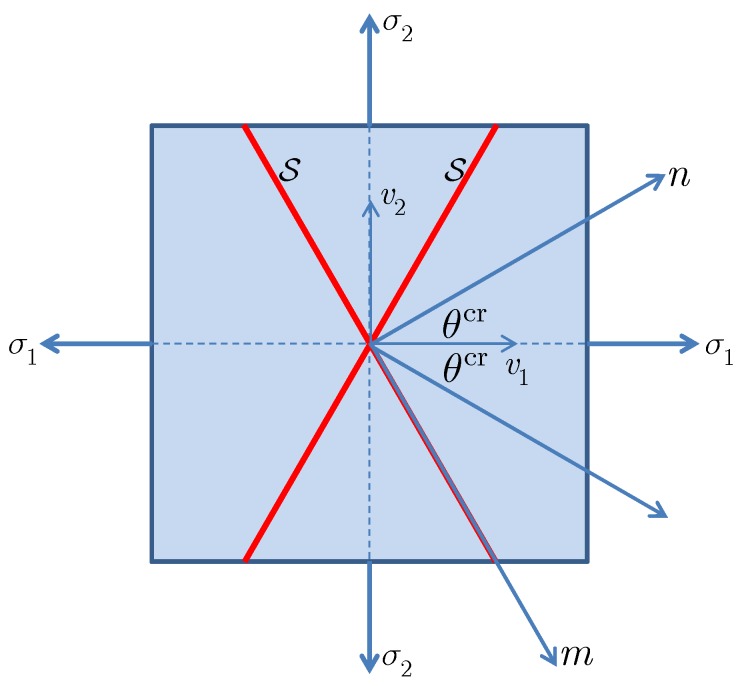
Definition of the discontinuity angle.

In particular, let us consider the 2D plane stress and plane strain conditions ($\sigma_{np}=\sigma_{mp}=0$). In such cases, the discontinuity orientation can be characterized by the inclination angle (anti-clockwise) $\theta^{\mathrm{cr}}\in \left[-\pi /2,\pi /2\right]$ between the normal vector n of the discontinuity and the principal vector $v_{1}$ of the tensor Λ; see Figure 4. Furthermore, the in-plane components $\left(\Lambda_{nn},\Lambda_{mm},\Lambda_{nm}\right)$ are given by:

(18a) $$\begin{array}{c} \Lambda_{nn}=\Lambda_{1}\cos^{2}\theta +\Lambda_{2}\sin^{2}\theta =\frac{\Lambda_{1}+\Lambda_{2}}{2}+\frac{\Lambda_{1}-\Lambda_{2}}{2}\cos\left(2\theta \right) \end{array}$$

(18b) $$\begin{array}{c} \Lambda_{mm}=\Lambda_{1}\sin^{2}\theta +\Lambda_{2}\cos^{2}\theta =\frac{\Lambda_{1}+\Lambda_{2}}{2}-\frac{\Lambda_{1}-\Lambda_{2}}{2}\cos\left(2\theta \right) \end{array}$$

(18c) $$\begin{array}{c} \Lambda_{nm}=\left(\Lambda_{1}-\Lambda_{2}\right)\cos\theta \sin\theta =\frac{\Lambda_{1}-\Lambda_{2}}{2}\sin\left(2\theta \right) \end{array}$$

for the in-plane principal values $\Lambda_{1}$ and $\Lambda_{2}$. As the out-of-plane constraint $\Lambda_{mp}\left(\theta^{\mathrm{cr}}\right)=0$ is automatically fulfilled, it follows from the condition $\Lambda_{mm}\left(\theta^{\mathrm{cr}}\right)=0$ that:

(19) $$\begin{array}{c} \sin^{2}\theta^{\mathrm{cr}}=-\frac{\Lambda_{2}}{\Lambda_{1}-\Lambda_{2}},\;\cos^{2}\theta^{\mathrm{cr}}=\frac{\Lambda_{1}}{\Lambda_{1}-\Lambda_{2}} \end{array}$$

where the in-plane principle values $\Lambda_{1}$ and $\Lambda_{2}$ of the tensor Λ, satisfying $\Lambda_{1}\geq 0$ and $\Lambda_{2}\leq 0$, are constrained by the conditions:

(20a) $$\begin{array}{cc} & \sigma_{3}\left(\theta^{\mathrm{cr}}\right)=\sigma_{pp}\left(\theta^{\mathrm{cr}}\right)=0\;\;\;\mathrm{Plane}\;\mathrm{stress} \end{array}$$

(20b) $$\begin{array}{cc} & \Lambda_{3}\left(\theta^{\mathrm{cr}}\right)=\Lambda_{pp}\left(\theta^{\mathrm{cr}}\right)=0\;\mathrm{Plane}\;\mathrm{strain} \end{array}$$

Note that in the plane stress state ($\sigma_{pp}=0$), the constraint $\Lambda_{pp}\left(\theta^{\mathrm{cr}}\right)=0$ makes no sense, and the cohesive relations (16a) always hold; see Remark 2 for more discussion.

**Remark** **1.** The above arguments also apply to a regularized discontinuity B with finite bandwidth b↛0. Note that the bandwidth b is a regularized parameter, which can be taken as small as desired. In such a case, the Kronecker-delta $\delta_{{}_{S}}$ is regularized by $\delta_{{}_{S}}\left(x\right)\approx \Xi \left(x\right)/b$, where the collocation function Ξ(x) is defined as Ξ(x)=1 for x∈B and Ξ(x)=0 otherwise; see [15,36].

**Remark** **2.** *To gain further insight into the above strain localization analysis, let us consider a regularized discontinuity B in the solid Ω with the following internal virtual work:*

(21) $$\begin{array}{c} \delta W=\int_{\Omega }\sigma :\delta \epsilon^{e}\;dV+\int_{B}\sigma :\delta \epsilon^{d}\;dV \end{array}$$

*For general 3D and plane strain cases, provided that the discontinuity angles $\theta^{\mathrm{cr}}$ satisfying the constraints* (17) *exist, the inelastic internal work localized in the discontinuity band B is expressed as:*

(22) $$\begin{array}{c} \int_{B}\sigma :\delta \epsilon^{d}\;dB=\int_{B}\left(\sigma_{nn}\delta \epsilon_{nn}^{d}+2\sigma_{nm}\delta \epsilon_{mm}^{d}+2\sigma_{np}\delta \epsilon_{nm}^{d}\right)\;dB=\int_{S}\left(t_{n}\delta w_{n}+t_{m}\delta w_{m}+t_{m}\delta w_{p}\right)\;dS \end{array}$$

*where the results* (16) *with the regularization relation $\omega =\bar{\omega }/b$ have been recalled. This is exactly the result for the elastic solid containing a frictional-cohesive crack. In the plane stress state ($\sigma_{pp}=0$), only the condition* (17a) *is necessary for guaranteeing the identity:*

(23) $$\begin{array}{c} \int_{B}\sigma :\delta \epsilon^{d}\;dB=\int_{B}\left(\sigma_{nn}\delta \epsilon_{nn}^{d}+2\sigma_{nm}\delta \epsilon_{nm}^{d}\right)\;dB=\int_{S}\left(t_{n}\delta w_{n}+t_{m}\delta w_{m}\right)\;dS \end{array}$$

*with the constraint $\Lambda_{pp}\left(\theta^{\mathrm{cr}}\right)=0$ being irrelevant. That is, regardless of the constraint $\Lambda_{pp}\left(\theta^{\mathrm{cr}}\right)=0$, strain localization also occurs in the plane stress condition, with the resulting discontinuity characterized by the cohesive relations* (16a).

## 3. Closed-Form Results

In this section, the strain localization analysis presented in Section 2.2 is applied to several classical 1−d damage models. Both the discontinuity orientation and the corresponding localized constitutive laws are given.

### 3.1. $J_{2}$ Damage Model

Let us first discuss the $J_{2}$ damage model. It is usually employed for the modeling of shear bands or slip lines in $J_{2}$ softening materials; see [46] for the details.

#### 3.1.1. Constitutive Relations

In the $J_{2}$ damage model, the stress and strain tensors are decomposed as:

(24) $$\begin{array}{c} \sigma =pI+s,\;\epsilon =\frac{1}{3}\epsilon_{v}I+e \end{array}$$

where $p=\frac{1}{3}\mathrm{tr}\sigma$ and s=σ−pI are the volumetric and deviatoric parts of the stress tensor σ, respectively; $\epsilon_{v}=\mathrm{tr}\epsilon$ and $e:=\epsilon -\frac{1}{3}\epsilon_{v}I$ represent the trace and the deviatoric part of the strain tensor ϵ, respectively.

While the volumetric behavior remains elastic, a scalar variable d∈[0,1] is introduced to characterize the deviatoric behavior. It then follows that:

(25) $$\begin{array}{c} p=K_{0}\epsilon_{v},\;s=\left(1-d\right)\bar{s}=\left(1-d\right)2G_{0}e \end{array}$$

where $\bar{s}=2G_{0}e$ is the effective deviatoric stress tensor; $K_{0}$ and $G_{0}$ are the elastic bulk and shear moduli of the material, respectively.

In this case, the constitutive relation (2) is particularized as:

(26) $$\begin{array}{c} \epsilon =C:\sigma ,\;C=\frac{1}{3K_{0}}I_{\mathrm{vol}}+\frac{1}{2\left(1-d\right)G_{0}}I_{\mathrm{dev}} \end{array}$$

for the volumetric and deviatoric projection operators:

(27) $$\begin{array}{c} I_{\mathrm{vol}}=\frac{1}{3}I⊗I,\;I_{\mathrm{dev}}=I-I_{\mathrm{vol}}=I-\frac{1}{3}I⊗I \end{array}$$

The damage compliance tensor $C^{d}$ and the resulting inelastic strain $\epsilon^{d}$ are then given by:

(28) $$\begin{array}{c} C^{d}=C-C_{0}=\frac{\omega }{2G_{0}}I_{\mathrm{dev}},\;\epsilon^{d}=C^{d}:\sigma =\frac{\omega }{2G_{0}}s=\omega \Lambda \end{array}$$

for the variable ω:=d/(1−d) and the characteristic tensor $\Lambda =s/(2G_{0})$.

The above $J_{2}$ damage model corresponds to the following Helmholtz free energy ψ:

(29) $$\begin{array}{c} \psi =\frac{1}{2}K_{0}\epsilon_{v}^{2}+\frac{1}{2}\left(1-d\right)2G_{0}e:e \end{array}$$

together with the energy dissipation inequality:

(30) $$\begin{array}{c} D=Y\cdot \dot{d}\geq 0\;Y=-\frac{\partial \psi }{\partial d}=G_{0}e:e=\frac{1}{4G_{0}}\bar{s}:\bar{s} \end{array}$$

for the energy release rate *Y*, conjugate to the damage variable *d*.

Additionally, a strain-based (or effective stress-based) damage criterion is introduced:

(31) $$\begin{array}{c} F_{\epsilon }\left(\epsilon ,r\right)=\epsilon_{\mathrm{eq}}\left(\epsilon \right)-r\leq 0 \end{array}$$

in terms of the equivalent strain $\epsilon_{\mathrm{eq}}\left(\epsilon \right)$ and the threshold *r*:

(32) $$\begin{array}{c} \epsilon_{\mathrm{eq}}\left(\epsilon \right)=\sqrt{\frac{1}{2}\bar{s}:\bar{s}}=\sqrt{{\bar{J}}_{2}},\;r=\max_{t\in [0,T]}\left(r_{0},\epsilon_{\mathrm{eq}}^{t}\right) \end{array}$$

for the second invariant ${\bar{J}}_{2}=\frac{1}{2}\bar{s}:\bar{s}$ of the effective deviatoric stress tensor $\bar{s}=2G_{0}e$. Upon damage loading, it follows from the consistency condition ${\dot{F}}_{\epsilon }\left(\epsilon ,r\right)=0$ that the threshold *r*, with the initial value $r_{0}$, represents the maximum value of $\epsilon_{\mathrm{eq}}$ ever reached.

In order to postulate the damage evolution law in the format (5), it is convenient to rewrite the above strain-based damage criterion as:

(33) $$\begin{array}{c} F_{\sigma }\left(\sigma ,q\right)=\sigma_{\mathrm{eq}}\left(\sigma \right)-q\leq 0 \end{array}$$

in terms of the following equivalent stress $\sigma_{\mathrm{eq}}$ and the corresponding softening variable q(r):

(34) $$\begin{array}{c} \sigma_{\mathrm{eq}}=\sqrt{\frac{1}{2}s:s}=\sqrt{J_{2}}=\left(1-d\right)\epsilon_{\mathrm{eq}},\;q=\left(1-d\right)r \end{array}$$

where $J_{2}=\frac{1}{2}s:s$ represents the second invariant of the deviatoric stress tensor s.

#### 3.1.2. Orientation of the Discontinuity

For the $J_{2}$ damage model with the characteristic tensor $\Lambda =s/(2G_{0})$, the conditions (17) upon strain localization become:

(35) $$\begin{array}{c} s_{mm}\left(\theta^{\mathrm{cr}}\right)=s_{pp}\left(\theta^{\mathrm{cr}}\right)=s_{mp}\left(\theta^{\mathrm{cr}}\right)=0 \end{array}$$

for the deviatoric stress components $\left(s_{mm},s_{pp},s_{mp}\right)$ on the discontinuity surface. Accordingly, the orientation angles $\theta^{\mathrm{cr}}$ can be solved in terms of the principal values $s_{i}\;\left(i=1,2,3\right)$ of the deviatoric stress tensor s.

In particular, for the 2D cases of plane stress and plane strain ($\sigma_{np}=\sigma_{mp}=0$), the condition $s_{mm}\left(\theta^{\mathrm{cr}}\right)=0$ gives:

(36) $$\begin{array}{c} \sin^{2}\theta^{\mathrm{cr}}=-\frac{s_{2}}{s_{1}-s_{2}},\;\cos^{2}\theta^{\mathrm{cr}}=\frac{s_{1}}{s_{1}-s_{2}} \end{array}$$

Furthermore, it follows from the constraint $s_{3}=s_{pp}=0$ that:

(37) $$\begin{array}{c} s_{1}=-s_{2}\;⟹\;\theta^{\mathrm{cr}}=\pm 45^{{}^{\circ}} \end{array}$$

That is, in 2D conditions strain localization of a $J_{2}$ damaging material with stress continuity can always occur along the discontinuity angle $\theta^{\mathrm{cr}}=\pm 45^{{}^{\circ}}$. Note that the above analytical result coincides with the numerical simulations [46].

#### 3.1.3. Localized Damage Model

Provided that the solution to the constraints (35) exists, it follows from the vanishing trace trs=0 that $s_{nn}=-\left(s_{mm}+s_{pp}\right)=0$ holds on the discontinuity surface with the normal $n(\theta^{\mathrm{cr}})$. Accordingly, the relation (14) becomes:

(38a) $$\begin{array}{c} \bar{\omega }\frac{s}{2G_{0}}={\left(w⊗n\right)}^{\mathrm{sym}}=w_{m}{\left(n⊗m\right)}^{\mathrm{sym}}+w_{p}{\left(n⊗p\right)}^{\mathrm{sym}} \end{array}$$

or, equivalently,

(38b) $$\begin{array}{c} s=\frac{2G_{0}}{\bar{\omega }}\left[w_{m}{\left(n⊗m\right)}^{\mathrm{sym}}+w_{p}{\left(n⊗p\right)}^{\mathrm{sym}}\right] \end{array}$$

The above frictional-cohesive relations can also be given straightforwardly from Equation (16):

(39) $$\begin{array}{c} w_{n}=\bar{\omega }\;\frac{s_{nn}}{2G_{0}}=0,\;w_{m}=\bar{\omega }\;\frac{s_{nm}}{G_{0}}=\bar{\omega }\;\frac{t_{m}}{G_{0}},\;w_{p}=\bar{\omega }\;\frac{s_{np}}{G_{0}}=\bar{\omega }\;\frac{t_{p}}{G_{0}} \end{array}$$

where the identities $s_{nm}=\sigma_{nm}=t_{m}$ and $s_{np}=\sigma_{np}=t_{p}$ have been considered.

With the frictional-cohesive relations (39) upon strain localization, the stress-based damage criterion (33) becomes:

(40) $$\begin{array}{c} f\left(t,q\right)=F_{\sigma }\left(\sigma ,q\right)=\frac{G_{0}}{\bar{\omega }}w_{\mathrm{eq}}-q=t_{\mathrm{eq}}-q\leq 0 \end{array}$$

or the alternative displacement jump-based one:

(41) $$\begin{array}{c} g\left(w,\kappa \right)=w_{\mathrm{eq}}-\kappa \leq 0,\;\kappa =\frac{\bar{\omega }}{G_{0}}q=\frac{1}{G_{0}}\frac{q-q_{0}}{\bar{H}} \end{array}$$

where $w_{\mathrm{eq}}$ and $t_{\mathrm{eq}}$ denote the equivalent displacement jump and traction, defined as:

(42) $$\begin{array}{c} w_{\mathrm{eq}}=\sqrt{w_{m}^{2}+w_{p}^{2}},\;t_{\mathrm{eq}}=\sqrt{t_{m}^{2}+t_{p}^{2}} \end{array}$$

Similarly to the strain-based threshold *r*, the displacement jump-like internal variable κ represents the maximum value of the equivalent displacement jump $w_{\mathrm{eq}}$ ever reached.

As expected, upon strain localization, the $J_{2}$ damage model is characterized by displacement jumps and tractions. That is, a pure mode-II discontinuity in the sense of fracture mechanics occurs in the $J_{2}$ damaging material.

### 3.2. Simó and Ju [41] Damage Model

Next, let us consider the Simó and Ju [41] damage model, which has been widely adopted in the literature. As will be shown, strain localization with stress continuity cannot always occur in any case.

#### 3.2.1. Constitutive Relations

The Simó and Ju [41] damage model is characterized by the following well-defined Helmholtz free energy:

(43) $$\begin{array}{c} \psi =\frac{1}{2}\left(1-d\right)\epsilon :E_{0}:\epsilon \end{array}$$

where $E_{0}=\lambda_{0}I⊗I+2\mu_{0}I$ represents the fourth-order material elasticity tensor, with $\lambda_{0}$ and $\mu_{0}$ being the Lamé constants. Standard arguments then yield:

(44) $$\begin{array}{c} \sigma =\frac{\partial \psi }{\partial \epsilon }=\left(1-d\right)\bar{\sigma },\;\bar{\sigma }=E_{0}:\epsilon \end{array}$$

or, equivalently,

(45) $$\begin{array}{c} \epsilon =C:\sigma ,\;C=\frac{1}{1-d}C_{0} \end{array}$$

Similarly, the damage compliance tensor $C^{d}$ and the resulting damage strain $\epsilon^{d}$ are characterized in the form (4):

(46) $$\begin{array}{c} C^{d}=C-C_{0}=\omega C_{0},\;\epsilon^{d}=C^{d}:\sigma =\omega C_{0}:\sigma =\omega \Lambda \end{array}$$

for the variable ω:=d/(1−d) and the characteristic tensor $\Lambda =C_{0}:\sigma$.

The above constitutive relations are constrained by the damage energy dissipation inequality:

(47) $$\begin{array}{c} D=Y\cdot \dot{d}\geq 0,\;Y=-\frac{\partial \psi }{\partial d}=\frac{1}{2}\epsilon :E_{0}:\epsilon =\frac{1}{2}\bar{\sigma }:C_{0}:\bar{\sigma } \end{array}$$

It is then possible to introduce the Beltrami strain-based damage criterion:

(48) $$\begin{array}{c} F_{\epsilon }\left(\epsilon ,r\right)=\epsilon_{\mathrm{eq}}\left(\epsilon \right)-r\leq 0 \end{array}$$

where the equivalent strain $\epsilon_{\mathrm{eq}}$ and the threshold *r* are defined as:

(49) $$\begin{array}{c} \epsilon_{\mathrm{eq}}\left(\epsilon \right)=\sqrt{M_{0}\left(\bar{\sigma }:C_{0}:\bar{\sigma }\right)},\;r=\max_{t\in [0,T]}\left(r_{0},\epsilon_{\mathrm{eq}}^{t}\right) \end{array}$$

for the longitudinal or constrained modulus $M_{0}=\lambda_{0}+2\mu_{0}$. Alternatively, for the damage evolution law (5), a stress-based failure criterion can be expressed as:

(50) $$\begin{array}{c} F_{\sigma }\left(\sigma ,q\right)=\sigma_{\mathrm{eq}}\left(\sigma \right)-q\leq 0 \end{array}$$

for the equivalent stress $\sigma_{\mathrm{eq}}$ and the corresponding stress-like internal variable *q*:

(51) $$\begin{array}{c} \sigma_{\mathrm{eq}}\left(\sigma \right)=\sqrt{M_{0}\left(\sigma :C_{0}:\sigma \right)}=\left(1-d\right)\epsilon_{\mathrm{eq}},\;q=\left(1-d\right)r \end{array}$$

Once the softening function q(r) is postulated, the damage variable *d* can be determined as shown later.

#### 3.2.2. Orientation of the Discontinuity

For the Simó and Ju [41] damage model, the characteristic tensor Λ is expressed as:

(52) $$\begin{array}{c} \Lambda =C_{0}:\sigma =\frac{1}{E_{0}}\left[\left(1+\nu_{0}\right)\sigma -\nu_{0}\mathrm{tr}\sigma \;I\right] \end{array}$$

so that the constraints (17) give:

(53) $$\begin{array}{c} \sigma_{mm}-\nu_{0}\left(\sigma_{nn}+\sigma_{pp}\right)=0,\;\sigma_{pp}-\nu_{0}\left(\sigma_{nn}+\sigma_{mm}\right)=0,\;\sigma_{mp}=0 \end{array}$$

With the stress components expressed in terms of the principal stresses $\sigma_{i}\;\left(i=1,2,3\right)$ and a set of characteristic angles θ, the discontinuity angles $\theta^{\mathrm{cr}}$ can be determined.

In particular, let us focus on the 2D plane stress and plane strain cases in which the identity $\sigma_{np}=\sigma_{mp}=0$ holds. For the plane stress state, the conditions (19) and (20a) give:

(54a) $$\begin{array}{c} \sigma_{mm}=\nu_{0}\sigma_{nn}\;⟹\;\cos\left(2\theta^{\mathrm{cr}}\right)=\frac{1-\nu_{0}}{1+\nu_{0}}\cdot \frac{\sigma_{1}+\sigma_{2}}{\sigma_{1}-\sigma_{2}} \end{array}$$

For the plane strain case (i.e., $\sigma_{3}=\sigma_{pp}\neq 0$), it follows from the conditions (19) and (20a) that:

(54b) $$\begin{array}{c} \sigma_{mm}=\sigma_{pp}=\frac{\nu_{0}}{1-\nu_{0}}\sigma_{nn}\;⟹\;\cos\left(2\theta^{\mathrm{cr}}\right)=\left(1-2\nu_{0}\right)\frac{\sigma_{1}+\sigma_{2}}{\sigma_{1}-\sigma_{2}} \end{array}$$

Note that the convention $\sigma_{1}>\sigma_{2}$ is assumed as usual. As can be seen, both results depend on Poisson’s ratio $\nu_{0}$ and coincide for a vanishing one.

#### 3.2.3. Localized Damage Model

Provided that the solution to the conditions (53) exists, it follows from the relation (14) that:

(55) $$\begin{array}{c} \sigma =\frac{1}{\bar{\omega }}E_{0}:{\left(w⊗n\right)}^{\mathrm{sym}} \end{array}$$

Accordingly, the frictional-cohesive relations are given by:

(56a) $$\begin{array}{c} t=n\cdot \sigma =\frac{1}{\bar{\omega }}Q_{0}\cdot w,\;w=\bar{\omega }Q_{0}^{-1}\cdot t \end{array}$$

or, in the component form,

(56b) $$\begin{array}{c} w_{n}=\bar{\omega }\Lambda_{nn}=\frac{\bar{\omega }}{M_{0}}\sigma_{nn},\;w_{m}=\bar{\omega }\Lambda_{nm}=\frac{\bar{\omega }}{G_{0}}\sigma_{nm},\;w_{p}=\bar{\omega }\Lambda_{np}=\frac{\bar{\omega }}{G_{0}}\sigma_{np} \end{array}$$

where the second-order elastic acoustic tensor $Q_{0}$ and its inverse $Q_{0}^{-1}$ are expressed as:

(57a) $$\begin{array}{cc} Q_{0}: & =n\cdot E_{0}\cdot n=M_{0}n⊗n+G_{0}\left(m⊗m+p⊗p\right) \end{array}$$

(57b) $$\begin{array}{cc} Q_{0}^{-1} & =\frac{1}{M_{0}}n⊗n+\frac{1}{G_{0}}\left(m⊗m+p⊗p\right) \end{array}$$

in the local (n,m,p) coordinate system.

With the relation (55), upon strain localization, the stress-based damage criterion (50) becomes:

(58) $$\begin{array}{c} f\left(t,q\right)=F_{\sigma }\left(\sigma ,q\right)=\frac{M_{0}}{\bar{\omega }}w_{\mathrm{eq}}-q=t_{\mathrm{eq}}-q\leq 0 \end{array}$$

or, the alternative form,

(59) $$\begin{array}{c} g\left(w,\kappa \right)=w_{\mathrm{eq}}-\kappa \leq 0,\;\kappa =\frac{\bar{\omega }}{M_{0}}q=\frac{1}{M_{0}}\frac{q-q_{0}}{\bar{H}} \end{array}$$

where the equivalent displacement jump $w_{\mathrm{eq}}$ and traction $t_{\mathrm{eq}}$ are expressed as:

(60a) $$\begin{array}{c} w_{\mathrm{eq}}=\sqrt{\frac{1}{M_{0}}\left(w\cdot Q_{0}\cdot w\right)}=\sqrt{w_{n}^{2}+\beta_{0}^{2}\left(w_{m}^{2}+w_{p}^{2}\right)} \end{array}$$

(60b) $$\begin{array}{cc} & t_{\mathrm{eq}}=\sqrt{M_{0}\left(t\cdot Q_{0}^{-1}\cdot t\right)}=\sqrt{t_{n}^{2}+\beta_{0}^{-2}\left(t_{m}^{2}+t_{p}^{2}\right)} \end{array}$$

for the coefficient $\beta_{0}=\sqrt{G_{0}/M_{0}}$.

The above localized cohesive law for the Simó and Ju [41] damage model was first derived in [22,24] where the discontinuity orientation was determined from the classical discontinuous bifurcation analysis rather than the Maxwell kinematic condition considered in this work.

### 3.3. Modified Damage Model

As can be seen from the cohesive relation (56b)${}_{1}$, the normal behavior upon strain localization is affected by the lateral deformation due to the non-vanishing Poisson’s ratio. This fact is inconsistent with the concept of a cohesive zone model in which the lateral deformations on the discontinuity surface are not considered. To reconcile this inconsistency, let us consider a modified Simó and Ju damage model.

#### 3.3.1. Constitutive Relations

In the modified damage model, the constitutive relations read:

(61) $$\begin{array}{c} \epsilon =C:\sigma ,\;C=C_{0}+C^{d} \end{array}$$

which gives the following damage strain tensor $\epsilon^{d}$:

(62) $$\begin{array}{c} \epsilon^{d}=C^{d}:\sigma =\omega \Lambda ,\;\Lambda ={\bar{C}}_{0}:\sigma \end{array}$$

where the compliance tensor $C^{d}$ is expressed as the form (4):

(63) $$\begin{array}{c} C^{d}=C-C_{0}=\omega {\bar{C}}_{0},\;{\bar{C}}_{0}=\mathrm{DIAG}\left[\frac{1}{E_{0}},\frac{1}{E_{0}},\frac{1}{E_{0}},\frac{1}{G_{0}},\frac{1}{G_{0}},\frac{1}{G_{0}}\right] \end{array}$$

for the variable ω:=d/(1−d). Here, the diagonal matrix ${\bar{C}}_{0}$ is the Voigt representation of a fourth-order reference compliance tensor, which is obtained from the elastic one $C_{0}$ by ignoring the lateral deformations caused by Poisson’s ratio. The explicit expressions of the resulting secant compliance C and stiffness E are given in Appendix A.

As these constitutive tensors are of major symmetry, the corresponding energy function ψ is defined as:

(64) $$\begin{array}{c} \psi \left(\epsilon ,d\right)=\frac{1}{2}\epsilon :E:\epsilon =\frac{1}{2}\sigma :C:\sigma =\frac{1}{2}\sigma :C_{0}:\sigma +\frac{d}{2\left(1-d\right)}\sigma :{\bar{C}}_{0}:\sigma \end{array}$$

Standard arguments give the constitutive relations (61) and the following energy dissipation inequality:

(65) $$\begin{array}{c} D=Y\cdot \dot{d}\geq 0,\;Y=-\frac{\partial \psi }{\partial d}=\frac{\sigma :{\bar{C}}_{0}:\sigma }{2{\left(1-d\right)}^{2}}=\frac{1}{2}\bar{\sigma }:{\bar{C}}_{0}:\bar{\sigma } \end{array}$$

for the effective stress tensor $\bar{\sigma }=\sigma /\left(1-d\right)$. Accordingly, the following strain-based damage criterion can be considered:

(66) $$\begin{array}{c} F_{\epsilon }\left(\epsilon ,r\right)=\epsilon_{\mathrm{eq}}\left(\epsilon \right)-r\leq 0 \end{array}$$

where the equivalent strain $\epsilon_{\mathrm{eq}}$ and the threshold *r* are given by:

(67) $$\begin{array}{c} \epsilon_{\mathrm{eq}}\left(\epsilon \right)=\sqrt{E_{0}\left(\bar{\sigma }:{\bar{C}}_{0}:\bar{\sigma }\right)},\;r=\max_{t\in [0,T]}\left(r_{0},\epsilon_{\mathrm{eq}}^{t}\right) \end{array}$$

Alternatively, the stress-based damage criterion is expressed as:

(68) $$\begin{array}{c} F_{\sigma }\left(\sigma ,q\right)=\sigma_{\mathrm{eq}}\left(\sigma \right)-q\leq 0 \end{array}$$

for the equivalent stress $\sigma_{\mathrm{eq}}$ and internal variable *q*:

(69) $$\begin{array}{c} \sigma_{\mathrm{eq}}=\sqrt{E_{0}\left(\sigma :{\bar{C}}_{0}:\sigma \right)},\;q=\left(1-d\right)r \end{array}$$

Note that the above equivalent strain and stress are subtly different from their counterparts in the Simó and Ju [41] damage model.

#### 3.3.2. Orientation of the Discontinuity

For the damage strain (62), the constraints (17) become:

(70) $$\begin{array}{c} \Lambda_{mm}\left(\theta^{\mathrm{cr}}\right)=\frac{\sigma_{mm}}{E_{0}}=0,\;\Lambda_{pp}\left(\theta^{\mathrm{cr}}\right)=\frac{\sigma_{pp}}{G_{0}}=0,\;\Lambda_{mp}\left(\theta^{\mathrm{cr}}\right)=\frac{\sigma_{mp}}{G_{0}}=0 \end{array}$$

With the stress components expressed in terms of the principal stresses $\sigma_{i}\;\left(i=1,2,3\right)$ and a set of characteristic angles θ, the discontinuity angles $\theta^{\mathrm{cr}}$ can thus be determined.

For the plane stress and plane stress conditions, both Constraints (20a) and (20b) coincide, and the last two conditions in Equation (70) are automatically fulfilled. It then follows from the remaining one $\Lambda_{mm}\left(\theta^{\mathrm{cr}}\right)=0$ that:

(71) $$\begin{array}{c} \sigma_{mm}\left(\theta^{\mathrm{cr}}\right)=0\;⟹\;\cos\left(2\theta^{\mathrm{cr}}\right)=\frac{\sigma_{1}+\sigma_{2}}{\sigma_{1}-\sigma_{2}} \end{array}$$

The above result applies for the biaxial tension-compression quadrant, i.e., $\sigma_{1}>0>\sigma_{2}$. For the case $\sigma_{1}>\sigma_{2}>0$, the discontinuity angle is given from $\cos(2\theta^{\mathrm{cr}})=1$, i.e., $\theta^{\mathrm{cr}}=0$. That is, the discontinuity orientation n coincides with the major principle vector $v_{1}$ of the stress tensor σ. Compared to the Simó and Ju [41] model, the discontinuity angle (71) does not depend on the elastic Poisson’s ratio. This is consistent with the conclusion drawn for stress-based material models [15,36].

#### 3.3.3. Localized Damage Model

Provided the solution to the conditions (53) exists, it follows from the relation (14) that:

(72) $$\begin{array}{c} \sigma =\frac{1}{\bar{\omega }}{\bar{E}}_{0}:{\left(w⊗n\right)}^{\mathrm{sym}} \end{array}$$

for the displacement jumps $w:={\left\{w_{n},w_{m},w_{p}\right\}}^{T}$. Accordingly, the frictional-cohesive tractions $t:={\left\{t_{n},t_{m},t_{p}\right\}}^{T}$ are given by:

(73a) $$\begin{array}{c} t=n\cdot \sigma ={\bar{E}}_{{}_{{}_{S}}}\cdot w,\;{\bar{E}}_{{}_{{}_{S}}}=\frac{1}{\bar{\omega }}{\bar{E}}_{0} \end{array}$$

or, inversely,

(73b) $$\begin{array}{c} w={\bar{C}}_{{}_{{}_{S}}}\cdot t,\;{\bar{C}}_{{}_{{}_{S}}}=E_{{}_{{}_{S}}}^{-1}=\bar{\omega }{\bar{C}}_{0} \end{array}$$

for the reference stiffness ${\bar{E}}_{0}$ and compliance ${\bar{C}}_{0}$ of the discontinuity:

(74a) $$\begin{array}{c} {\bar{E}}_{0}=n\cdot {\bar{E}}_{0}\cdot n=E_{0}n⊗n+G_{0}\left(m⊗m+p⊗p\right) \end{array}$$

(74b) $$\begin{array}{c} {\bar{C}}_{0}={\bar{E}}_{0}^{-1}=\frac{1}{E_{0}}n⊗n+\frac{1}{G_{0}}\left(m⊗m+p⊗p\right) \end{array}$$

Note that in 2D plane stress and plane strain conditions, the above frictional-cohesive relations also apply with a vanishing out-of-plane jump $w_{p}=0$ (or, equivalently, $t_{p}=0$).

After calling for the relation (72) and performing some straightforward manipulations, upon strain localization, the stress-based damage criterion (50) becomes:

(75) $$\begin{array}{c} f\left(t,q\right)=F_{\sigma }\left(\sigma ,q\right)=\frac{E_{0}}{\bar{\omega }}w_{\mathrm{eq}}-q=t_{\mathrm{eq}}-q\leq 0 \end{array}$$

or, equivalently,

(76) $$\begin{array}{c} g\left(w,\kappa \right)=w_{\mathrm{eq}}-\kappa \leq 0,\;\kappa =\frac{\bar{\omega }}{E_{0}}q=\frac{1}{E_{0}}\frac{q-q_{0}}{\bar{H}} \end{array}$$

where the equivalent displacement jump $w_{\mathrm{eq}}$ and traction $t_{\mathrm{eq}}$ are expressed as:

(77) $$\begin{array}{c} w_{\mathrm{eq}}=\sqrt{w_{n}^{2}+\beta_{0}^{2}\left(w_{m}^{2}+w_{p}^{2}\right)},\;t_{\mathrm{eq}}=\sqrt{t_{n}^{2}+\beta_{0}^{-2}\left(t_{m}^{2}+t_{p}^{2}\right)} \end{array}$$

for the material property $\beta_{0}=\sqrt{G_{0}/E_{0}}$.

Compared to the frictional-cohesive relations (56) resulting from the Simó and Ju [41] damage model, the longitudinal modulus $M_{0}$ in Equation (57) is replaced here by Young’s modulus $E_{0}$. This modification is consistent with the concept of a frictional-cohesive crack model in which only the strain components acting on the discontinuity surface are accounted for [47]. Moreover, upon the assumption of a continuous elastic strain field $\epsilon^{e}\left(x\right)$ across the discontinuity, it can be regularized as the smeared crack model discussed in [38,48]; see Appendix B for more details. The above facts justify the above modified Simó and Ju damage model, which will be addressed in the numerical context elsewhere.

## 4. Numerical Verification

In this section, the analytical results presented in Section 3 are numerically verified. Due to the poor resolution upon strain localization, the irreversible displacement-based finite element method is not sufficient for this purpose. In order to circumvent this difficulty, the mixed stabilized strain/displacement ϵ−u finite element method, recently developed by Cervera et al. [39] for elasticity and extended to isotropic damage materials [40,49], is considered.

### 4.1. Mixed Stabilized Strain/Displacement Element

The aforesaid mixed stabilized strain/displacement element is briefly recalled in a secant format appropriate for the continuum damage models discussed in this work. The mechanical behavior of the solid body Ω is described by the compatibility of deformations and the equilibrium of body forces:

(78a) $$\begin{array}{c} -\epsilon +\nabla^{\mathrm{sym}}u=0 \end{array}$$

(78b) $$\begin{array}{c} \nabla \cdot \sigma +b^{*}=0 \end{array}$$

where u is the displacement vector, ϵ is the strain tensor, σ represents the stress tensor, $\nabla^{\mathrm{sym}}$ and ∇·(·) are the adjoint symmetric gradient and the divergence operators, respectively; $b^{*}$ is the body force vector. The strain and stress tensors are linked by the secant constitutive relations (1) with appropriate damage evolution laws. The strong form of the boundary value problem is completed by imposing proper traction and displacement boundary conditions on ∂Ω.

After pre-multiplying the secant stiffness tensor E, the strong form reads:

(79a) $$\begin{array}{c} -E:\epsilon +E:\nabla^{\mathrm{sym}}u=0 \end{array}$$

(79b) $$\begin{array}{c} \nabla \cdot \left(E:\epsilon \right)+b^{*}=0 \end{array}$$

for the unknowns fields of total strains ϵ and displacements u. Introducing the test function $\gamma \in G\subset {\left[L^{2}\left(\Omega \right)\right]}^{\dim}$ for strains and $v\in V\subset {\left[H^{1}\left(\Omega \right)\right]}^{\dim}$ for displacements and applying Gauss’s divergence theorem to the strong form yields the following weak form of the mixed problem:

(80a) $$\begin{array}{c} -\int_{\Omega }\gamma :E:\epsilon \;dV+\int_{\Omega }\gamma :E:\nabla^{\mathrm{sym}}u\;dV=0\;\forall \gamma \in G \end{array}$$

(80b) $$\begin{array}{c} \int_{\Omega }\nabla^{\mathrm{sym}}v:\left(E:\epsilon \right)\;dV=F\left(v\right)\;\forall v\in V \end{array}$$

where F(v) represents the work done by boundary tractions $t^{*}$ and body forces $b^{*}$. Note that this weak form is symmetric.

The discrete version of the mixed weak form is obtained by substituting the unknown fields with their finite element interpolation counterparts:

(81) $$\begin{array}{c} \epsilon \to \epsilon_{h}=\sum_{i=1}^{n_{pts}}\gamma_{h}^{\left(i\right)}\epsilon_{h}^{\left(i\right)}\;\gamma_{h}\in G_{h};\;u\to u_{h}=\sum_{i=1}^{n_{pts}}v_{h}^{\left(i\right)}u_{h}^{\left(i\right)}\;v_{h}\in V_{h} \end{array}$$

where $\epsilon_{h}$ and $u_{h}$ are the nodal degrees of freedom; $\gamma_{h}$ and $v_{h}$ are the discrete test functions for the strains and displacements pertaining to the spaces $G_{h}$ and $V_{h}$, respectively, the discrete counterparts of G and V.

As implied by the *Inf-Sup*condition [50], equal interpolations for strains and displacements are bound to be unstable. To overcome this issue, a stabilization procedure needs be introduced by modifying the discrete variational form with numerical stability while maintaining consistency. For the stabilized problem [39,40] derived the following system of equations:

(82a) $$\begin{array}{c} -\left(1-\tau_{\epsilon }\right)\int_{\Omega }\gamma_{h}:E:\left(\epsilon_{h}-\nabla^{\mathrm{sym}}u_{h}\right)\;dV=0\;\forall \gamma_{h}\in G_{h} \end{array}$$

(82b) $$\begin{array}{c} \int_{\Omega }\nabla^{\mathrm{sym}}v_{h}:E:{\tilde{\epsilon }}_{h}\;dV=F\left(v_{h}\right)\;\forall v_{h}\in V_{h} \end{array}$$

where the stabilized discrete strain field ${\tilde{\epsilon }}_{h}$ is a blending of the continuous and discontinuous strain fields $\left(\epsilon_{h},\nabla^{\mathrm{sym}}u_{h}\right)$ weighted by the stabilization parameter $\tau_{\epsilon }$:

(83) $$\begin{array}{c} {\tilde{\epsilon }}_{h}=\left(1-\tau_{\epsilon }\right)\epsilon_{h}+\tau_{\epsilon }\nabla^{\mathrm{sym}}u_{h}\;\mathrm{with}\;\tau_{\epsilon }=c_{\epsilon }\frac{h}{L_{0}} \end{array}$$

for the arbitrary positive parameter $c_{\epsilon }$, the representative mesh size *h* and the characteristic length $L_{0}$ of the problem. Note that the above stabilized formulation is consistent with the original discrete weak form since, with converging values of the unknowns $\epsilon_{h}$ and $u_{h}$, the contribution of the stabilization terms (those multiplied by $\tau_{\epsilon }$) vanishes.

### 4.2. Numerical Results

The above mixed stabilized strain-displacement element is then applied to analyses of strain localization. As the $J_{2}$ damage model has already been thoroughly verified in [46], only the Simó and Ju [41] damage model and the modified one are presented here.

**Figure 5:**
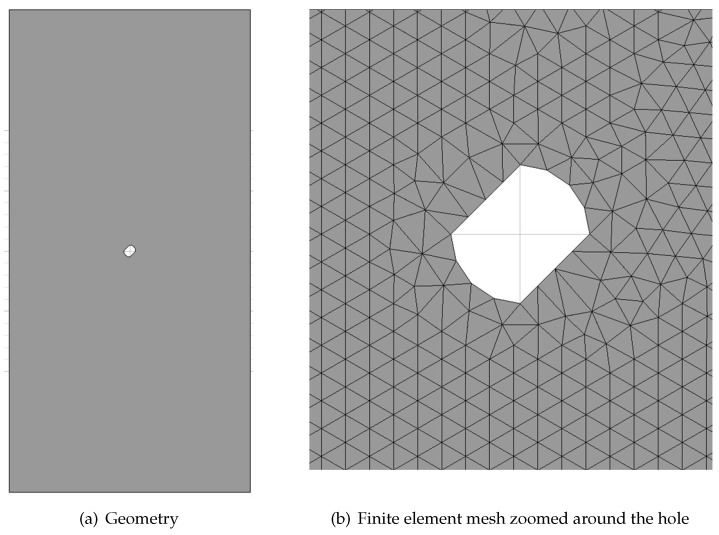
Uniaxial tension of a perforated strip: problem setting.

The example is a 2D singly perforated strip loaded in uniaxial tension by stretching via imposed vertical displacements at the top and bottom ends; horizontal movement is not restrained. Both plane strain and plane stress conditions are considered and compared. Figure 5a depicts the geometry of the problem with dimensions 20 m × 40 m ×1 m (width × height × thickness). An imperfection is introduced with a slanted perforation of diameter D=1 m such that symmetric solutions are excluded.

In all simulations, the exponential softening curve (6b) is considered and regularized according to the crack band theory [12], with the softening parameter *H* determined as:

(84) $$\begin{array}{c} H=-\frac{l_{\mathrm{ch}}}{L_{H}-0.5l_{\mathrm{ch}}} \end{array}$$

where $l_{\mathrm{ch}}$ and $L_{H}:=E_{0}G_{F}/q_{0}^{2}$ represent the localization and Griffith’s characteristic lengths, respectively. In the mixed formulation, $l_{\mathrm{ch}}=2h$, with *h* being the finite element mesh size.

The following material properties are assumed: Young’s modulus $E_{0}=10$ MPa, uniaxial failure stress $q_{0}=E/1000$ = 10 KPa and fracture energy is $G_{F}=500$ J/m${}^{2}$. Poisson’s ratio $\nu_{0}$ takes different values for comparison purposes.

The mesh used in the analyses consists of 45,750 mixed P1P1triangles (23,183 nodes) as shown in Figure 5b, with an average mesh size of h=0.20 m. This level of refinement ensures convergence of the numerical simulation and allows comparison with the analytical solution. The mesh is fully unstructured. All analyses are performed on the same mesh.

Loading is applied by imposed vertical displacements at both ends of the strip. The Newton–Raphson method is used to solve the nonlinear system of equations arising from the spatial and temporal discretization of the problem. An automatic procedure is used to decide the step size, and about 200 steps are necessary to complete the analyses. Convergence of a time step is attained when the ratio between the norms of the residual and the total forces is lower than $10^{-3}$.

Calculations are performed with an enhanced version of the finite element program COMET [51], developed by the authors at the International Center for Numerical Methods in Engineering (CIMNE). Pre- and post-processing is done with GiD, also developed at CIMNE [52].

#### 4.2.1. Simó and Ju [41] Damage Model

Let us first discuss the Simó and Ju [41] damage model. For the considered uniaxial stress state ($\sigma_{1}>0$ and $\sigma_{2}=0$), it follows from the closed-form results (54) that:

(85) $$\begin{array}{c} \cos\left(2\theta^{\mathrm{cr}}\right)=\left\{\begin{array}{cc} \frac{1-\nu_{0}}{1+\nu_{0}} & \;\mathrm{Plane}\;\mathrm{stress}\\ 1-2\nu_{0} & \;\mathrm{Plane}\;\mathrm{strain} \end{array}\right. \end{array}$$

The resulting discontinuity angles $\theta^{\mathrm{cr}}$ for various Poisson’s ratio $\nu_{0}$ are summarized in Table 1.

**Figure 6:**
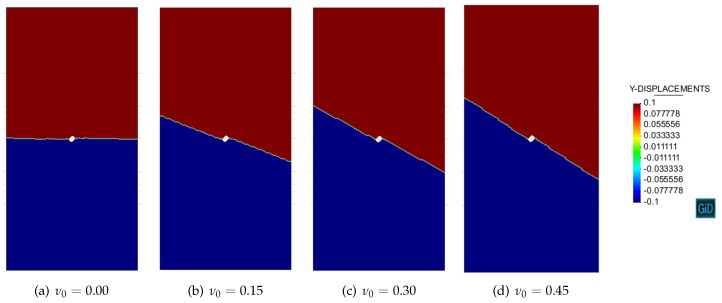
Uniaxial tension of a perforated strip: Simó and Ju [41] damage model in the plane stress condition.

**Figure 7:**
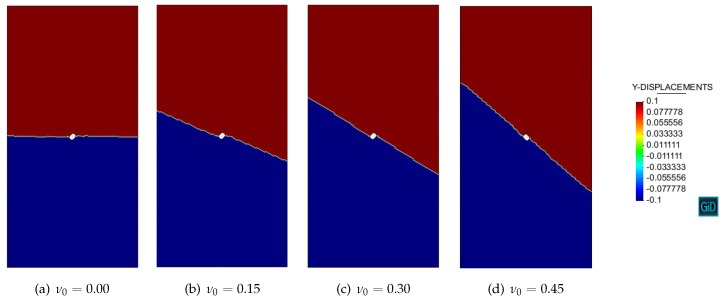
Uniaxial tension of a perforated strip: Simó and Ju [41] damage model in the plane strain condition.

Figure 6 and Figure 7 show the numerical contours of vertical displacements with respect to Simó and Ju [41] damage model in the plane stress and plane strain conditions. Strain localization is manifested by the discontinuous displacement field. The numerical discontinuity angles $\theta^{\mathrm{cr}}$ are then determined as in Table 1.

As can be seen, the analytical results agree almost exactly with the numerical ones, verifying the validity of both the novel strain localization analysis presented in this work and the mixed stabilized finite element method proposed in [39,40]. Slight differences are attributed to the unavoidable perturbation of the hole and to the boundary effects.

**Figure 8:**
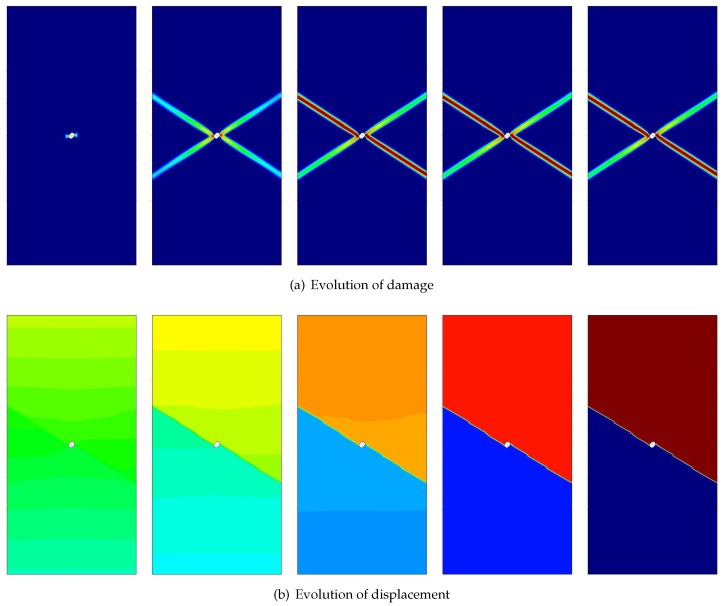
Uniaxial tension of a perforated strip: evolution of damage and vertical displacements for the Simó and Ju [41] model in the case of plane strain with Poisson’s ratio $\nu_{0}=0.30$.

**Figure 9:**
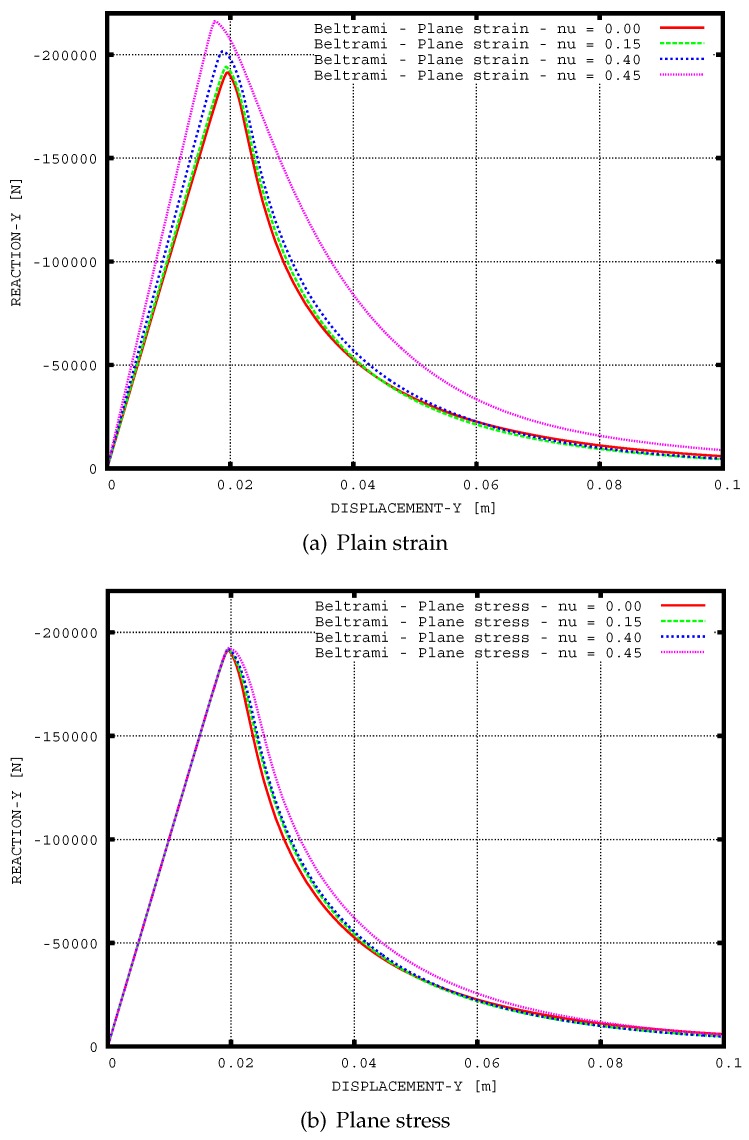
Uniaxial tension of a perforated strip: load versus displacement curves for the Simó and Ju [41] model.

For illustration, the evolution of damage and vertical displacements for the Simó and Ju [41] model in the case of plane strain with Poisson’s ratio $\nu_{0}=0.30$ is shown in Figure 8. Furthermore, load-displacement curves for the Simó and Ju [41] model in the conditions of plane strain and plane stress with various Poisson’s ratio $\nu_{0}=0.0,0.15,0.30$ and 0.45 are given in Figure 9.

#### 4.2.2. Modified Simó and Ju Damage Model

For the modified Simó and Ju damage model, the discontinuity angle $\theta^{\mathrm{cr}}$ given by the analytical results (71) is independent of Poisson’s ratio $\nu_{0}$. In particular, for the uniaxial stress state, it follows that:

(86) $$\begin{array}{c} \cos\theta^{\mathrm{cr}}=1\;⟹\;\theta^{\mathrm{cr}}=0 \end{array}$$

for both plane stress and plane strain states.

**Figure 10:**
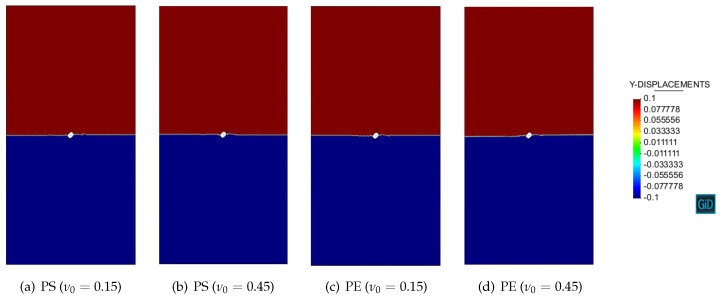
Uniaxial tension of a perforated strip: Modified Simó and Ju damage model in the plane stress (PS) and plane strain (PE) conditions.

Figure 10 shows the numerical contours of vertical displacements obtained from the modified Simó and Ju [41] damage model in the plane stress and plane strain conditions. As predicted from the analytical result (86), the displacement field exhibits a jump across the horizontal discontinuity surface, normal to the principal major stress, i.e., $\theta^{\mathrm{cr}}=0$. The novel strain localization analysis and the mixed stabilized finite element method are verified again.

## 5. Conclusions

This paper presents Maxwell’s kinematics-based strain localization analysis of frictional-cohesive materials characterized by strain-based damage models of total form, extending our recent work for stress-based ones of rate type. In such models, the (inelastic) damage strains are characterized by a bounded characteristic tensor and an unbounded variable related to the damage scalar. For strain localization to occur, Maxwell’s kinematics of a strong (or regularized) discontinuity has to be reproduced by the inelastic damage strains. This kinematic constraint yields a set of nonlinear equations from which the discontinuity orientation and localized frictional-cohesive relations of damage type are derived consistently.

Two classical isotropic damage models, i.e., the $J_{2}$ model and the Simó and Ju [41] one, are then analyzed. In particular, upon strain localization, the $J_{2}$ damaging solid is characterized by a pure mode-II discontinuity in which only the tangential tractions (or displacement jumps) are activated. Comparatively, a mixed-mode discontinuity occurs in the Simó and Ju [41] isotropic damaging solid. Furthermore, the localization angles and the resulting cohesive model depend explicitly on Poisson’s ratio. That is, the lateral deformations, not acting on the discontinuity surface, affect heavily strain localization, inconsistent with classical frictional-crack models in which the strain and stress triaxiality is not accounted for.

To remove the above inconsistency, we further proposed a modified Simó and Ju damage model. Its strain localization analysis naturally yields a consistent damage-type cohesive crack model, with the discontinuity orientation independent of the material elastic property (i.e., Poisson’s ratio). It is found that, on the one hand, the discontinuous version of the cohesive crack model is formally identical, but not equivalent to that first proposed in [7] and widely applied in the literature. On the other hand, the regularized version recovers the classical smeared crack model [47,48,53] upon the assumption of a continuous stress field across the discontinuity band.

The analytical results are numerically-verified by the mixed stabilized finite element method, regarding a singly perforated plate under uniaxial tension. Remarkably, for all of the damage models discussed in this work, the numerically-obtained localization angles agree almost exactly with the closed-form results. This agreement, on the one hand, consolidates the strain localization analysis based on Maxwell’s kinematics and, on the other hand, illustrates versatility of the mixed stabilized finite element method.

Finally, so far, only the material models with associated evolution laws have been considered in Maxwell’s kinematics-based strain localization analysis. The extension to non-associated cases will be the forthcoming work.

## Figures and Tables

**Table 1:** Comparison between the analytically- and numerically-obtained discontinuity angles $\theta^{\mathrm{cr}}$ for the Simó and Ju [41] damage model.

Stress State	$\nu_{0}=0.00$		$\nu_{0}=0.15$		$\nu_{0}=0.30$		$\nu_{0}=0.45$	
	Analytical	Numerical	Analytical	Numerical	Analytical	Numerical	Analytical	Numerical
Plane stress	$0.00^{{}^{\circ}}$	$0.00^{{}^{\circ}}$	$21.17^{{}^{\circ}}$	$20.75^{{}^{\circ}}$	$28.71^{{}^{\circ}}$	$28.69^{{}^{\circ}}$	$33.85^{{}^{\circ}}$	$33.13^{{}^{\circ}}$
Plane strain	$0.00^{{}^{\circ}}$	$0.00^{{}^{\circ}}$	$22.79^{{}^{\circ}}$	$22.31^{{}^{\circ}}$	$33.21^{{}^{\circ}}$	$32.27^{{}^{\circ}}$	$42.13^{{}^{\circ}}$	$41.48^{{}^{\circ}}$

**Figure B1:**
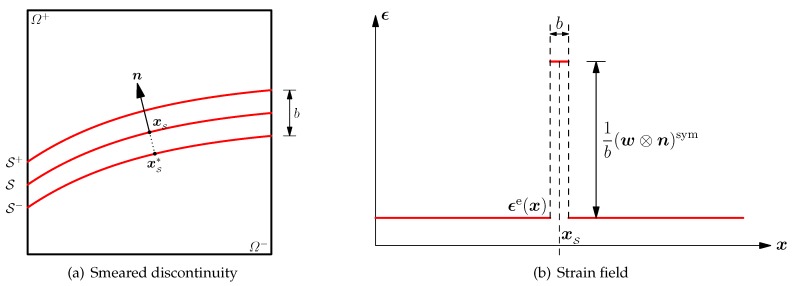
Smeared discontinuity and the resulting discontinuous strain field in an elastic solid.

## Appendix A. Modified Damage Model: Compliance and Stiffness Matrices

For the modified damage model presented in Section 3.3, the secant compliance is expressed in matrix form as:

(A1) $$\begin{array}{cc} C & =\frac{1}{E_{0}}\left[\begin{array}{cccccc} \frac{1}{1-d} & -\nu_{0} & -\nu_{0} & 0 & 0 & 0\\ -\nu_{0} & \frac{1}{1-d} & -\nu_{0} & 0 & 0 & 0\\ -\nu_{0} & -\nu_{0} & \frac{1}{1-d} & 0 & 0 & 0\\ 0 & 0 & 0 & \frac{2\left(1+\nu_{0}\right)}{1-d} & 0 & 0\\ 0 & 0 & 0 & 0 & \frac{2\left(1+\nu_{0}\right)}{1-d} & 0\\ 0 & 0 & 0 & 0 & 0 & \frac{2\left(1+\nu_{0}\right)}{1-d} \end{array}\right] \end{array}$$

The above expression applies to general 3D and plane strain conditions. For the case of plane stress, it follows that:

(A2) $$\begin{array}{c} C=\frac{1}{E_{0}}\left[\begin{array}{ccc} \frac{1}{1-d} & -\nu_{0} & 0\\ -\nu_{0} & \frac{1}{1-d} & 0\\ 0 & 0 & \frac{2\left(1+\nu_{0}\right)}{1-d} \end{array}\right] \end{array}$$

Note that under uniaxial tension, the nominal Poisson’s ratio predicted by the modified damage model is given by:

(A3) $$\begin{array}{c} \nu =-\frac{\epsilon_{22}}{\epsilon_{11}}=\left(1-d\right)\nu_{0} \end{array}$$

As expected, it decreases with increasing axial deformations. Contrariwise, in the Simó and Ju [41] isotropic damage model, the lateral deformations always increase in such a way that the nominal Poisson’s ratio remains constant.

## Appendix B. Smeared Crack Model: Compliance and Stiffness Matrices

As clarified in [14,15], the cohesive crack model for a strong discontinuity with a vanishing bandwidth b→0 is consistent with a regularized one for a discontinuity band with a finite width b↛0; see Figure B1a. For such a regularized discontinuity, an inelastic deformation vector e:=w/b can be introduced such that:

(A4a) $$\begin{array}{c} e_{n}:=\frac{w_{n}}{b}=\frac{\omega }{E_{0}}\sigma_{nn},\;e_{m}:=\frac{w_{m}}{b}=\frac{\omega }{G_{0}}\sigma_{nm},\;e_{p}:=\frac{w_{p}}{b}=\frac{\omega }{G_{0}}\sigma_{np} \end{array}$$

or, equivalently,

(A4b) $$\begin{array}{c} e=\omega {\bar{C}}_{0}\cdot t={\bar{C}}_{{}_{S}}\cdot t,\;{\bar{C}}_{{}_{S}}=\omega {\bar{C}}_{0} \end{array}$$

for the damage variable $\omega :=d/\left(1-d\right)=\bar{\omega }/b$ and the compliance ${\bar{C}}_{{}_{S}}$ of the regularized discontinuity.

In both the frictional-cohesive relations and the regularized counterparts, the kinematic unknowns related to the discontinuity, i.e., the displacement jump w or the inelastic deformation vector e, are determined through the statement of the traction continuity condition, i.e.,

(A5) $$\begin{array}{c} t=\sigma_{{}_{S}}^{+}\cdot n=\sigma_{{}_{S}}^{-}\cdot n \end{array}$$

where $\sigma_{{}_{S}}^{+}$ and $\sigma_{{}_{S}}^{-}$ represent the bulk stresses “ahead of” and “behind” the discontinuity S.

Alternatively, as depicted in Figure B1b, if the stresses are assumed to be continuous across the discontinuity, i.e., $\sigma_{{}_{S}}^{+}=\sigma_{{}_{S}}^{-}=\sigma$, traction continuity t=σ·n follows automatically, and no specific kinematic unknowns for the discontinuity are necessary. In this case, it follows from the regularized cohesive relations (A4) that:

(A6) $$\begin{array}{c} \epsilon^{d}={\left(e⊗n\right)}^{\mathrm{sym}}={\left[\left({\bar{C}}_{{}_{S}}\cdot t\right)⊗n\right]}^{\mathrm{sym}}=C^{d}:\sigma \end{array}$$

for the fourth-order damage compliance $C^{d}$:

(A7) $$\begin{array}{c} C^{d}=n⊗{\bar{C}}_{{}_{S}}⊗n={\left({\bar{C}}_{{}_{S}}\;\underline{\bar{⊗}}\;N\right)}^{\mathrm{sym}} \end{array}$$

The resulting stress versus strain relation become:

(A8) $$\begin{array}{c} \epsilon =\epsilon^{e}+\epsilon^{d}=C:\sigma \end{array}$$

where the compliance tensor C is expressed as:

(A9) $$\begin{array}{c} C=C_{0}+C^{d}=C_{0}+{\left({\bar{C}}_{{}_{S}}\;\underline{\bar{⊗}}\;N\right)}^{\mathrm{sym}} \end{array}$$

for the second-order geometric tensor N=n⊗n.

In matrix notation, the compliance C of the smeared crack model is given by:

(A10) $$\begin{array}{c} C=\frac{1}{E_{0}}\left[\begin{array}{cccccc} \frac{1}{1-d} & -\nu_{0} & -\nu_{0} & 0 & 0 & 0\\ -\nu_{0} & 1 & -\nu_{0} & 0 & 0 & 0\\ -\nu_{0} & -\nu_{0} & 1 & 0 & 0 & 0\\ 0 & 0 & 0 & \frac{2\left(1+\nu_{0}\right)}{1-d} & 0 & 0\\ 0 & 0 & 0 & 0 & \frac{2\left(1+\nu_{0}\right)}{1-d} & 0\\ 0 & 0 & 0 & 0 & 0 & 2\left(1+\nu_{0}\right) \end{array}\right] \end{array}$$

which applies to general 3D and plane strain conditions. In the case of plane stress, it follows that:

(A11) $$\begin{array}{cc} C & =\frac{1}{E_{0}}\left[\begin{array}{ccc} \frac{1}{1-d} & -\nu_{0} & 0\\ -\nu_{0} & 1 & 0\\ 0 & 0 & \frac{2\left(1+\nu_{0}\right)}{1-d} \end{array}\right] \end{array}$$

As can be seen, upon strain localization, the damage affects only the behavior normal to the discontinuity surface, while the one parallel to it remains elastic. This idea dates back to the classical smeared crack models [53,54,55,56]; see also the reviews [38,48].
